# Supplementary material for: Use of dual-flow bioreactor to develop a simplified model of nervous-cardiovascular systems crosstalk: A preliminary assessment
Source: PLoS One. 2020 Nov 30;15(11):e0242627. doi: 10.1371/journal.pone.0242627 (PMC7703955; doi:10.1371/journal.pone.0242627)
Supplement: Attachment — Submitted filename: Response to Reviewers 1.docx [file pone.0242627.s001.docx]

Dr. Laura Calvillo PhD

Istituto Auxologico Italiano IRCCS, Cardiology Research Laboratory, Department of Cardiovascular, Neural and Metabolic Sciences, S.Luca Hospital, Milan, Italy.

Via Magnasco 2-20149-Milan-Italy

l.calvillo@auxologico.it

To the Editor-in-Chief

Plos One October 19^th^, 2020

Dear Dr. Heber,

attached please find the required documents including the revised version (both with Tracked Changes and clean ) of our paper entitled:

“Use of dual-flow bioreactor to develop a simplified model of nervous-cardiovascular systems crosstalk: a preliminary assessment” by Marchesi N., Barbieri A., Fahmideh F., Govoni S., Ghidoni A., Parati G., Vanoli E., Pascale A., and myself. We have carefully considered all the constructive points of criticism raised by the reviewers, and we have modified our paper accordingly. We believe that, with these changes, our paper has improved significantly.

In particular, we have modified part of the introduction and discussion paragraphs. Moreover, to better describe the crosstalk between the two cell lines as suggested by Reviewer 2, we have performed an additional experiment, measuring VEGF release in the medium of HCASMC. Material and methods and results paragraphs have been modified accordingly, with new data and an additional figure (Figure 8) added in the manuscript. A real photo with cells on the dish has been provided (new Fig. 2) and Fig 1, describing the setting, has been improved.

We also attach the pdf of the book chapter: *Govoni S. (2020) Psychiatric and Neurological Effects of Cardiovascular Drugs. In: Govoni S., Politi P., Vanoli E. (eds) Brain and Heart Dynamics. Springer, Cham.* [*https://doi.org/10.1007/978-3-319-90305-7_46-1*](https://doi.org/10.1007/978-3-319-90305-7_46-1), as required by Reviewer 1.

Finally, as required by Editors, we have amended the title on the online submission form so that it is identical to the manuscript title, and we have provided the original uncropped and unadjusted images underlying all blot or gel results reported in the submission’s figures as supplement information named “S1_raw_images”, available at public repository Zenodo:

<https://doi.org/10.5281/zenodo.4106227>

On behalf of the co-authors, I declare that the manuscript has not been published nor submitted for publication elsewhere, and that all authors made substantial contribution to the concept, design, drafting and critical revision of the manuscript. All authors provided final approval of the manuscript and declared potential conflicts of interest, whenever present, which are listed in the manuscript submitted.

We wish to thank you and the Editorial Staff for the time you will devote to evaluate our paper. We hope that, with the changes we have made, you might find our revised work suitable for publication in PLOS ONE.

Kindest Regards

Sincerely,

Laura Calvillo on behalf of all authors.
