# Supplementary material for: Use of dual-flow bioreactor to develop a simplified model of nervous-cardiovascular systems crosstalk: A preliminary assessment
Source: PLoS One. 2020 Nov 30;15(11):e0242627. doi: 10.1371/journal.pone.0242627 (PMC7703955; doi:10.1371/journal.pone.0242627)
Supplement: Attachment — Submitted filename: Response to Reviewers_2.docx [file pone.0242627.s002.docx]

# Response to Reviewers

Comments to the Author

1. If the authors have adequately addressed your comments raised in a previous round of review and you feel that this manuscript is now acceptable for publication, you may indicate that here to bypass the “Comments to the Author” section, enter your conflict of interest statement in the “Confidential to Editor” section, and submit your "Accept" recommendation.

Reviewer #2: All comments have been addressed

*We thank the Reviewer for the time dedicated to our work*

2. Is the manuscript technically sound, and do the data support the conclusions?

The manuscript must describe a technically sound piece of scientific research with data that supports the conclusions. Experiments must have been conducted rigorously, with appropriate controls, replication, and sample sizes. The conclusions must be drawn appropriately based on the data presented.

Reviewer #2: Yes

3. Has the statistical analysis been performed appropriately and rigorously?

Reviewer #2: Yes

4. Have the authors made all data underlying the findings in their manuscript fully available?

The PLOS Data policy requires authors to make all data underlying the findings described in their manuscript fully available without restriction, with rare exception (please refer to the Data Availability Statement in the manuscript PDF file). The data should be provided as part of the manuscript or its supporting information, or deposited to a public repository. For example, in addition to summary statistics, the data points behind means, medians and variance measures should be available. If there are restrictions on publicly sharing data—e.g. participant privacy or use of data from a third party—those must be specified.

Reviewer #2: Yes

5. Is the manuscript presented in an intelligible fashion and written in standard English?

PLOS ONE does not copyedit accepted manuscripts, so the language in submitted articles must be clear, correct, and unambiguous. Any typographical or grammatical errors should be corrected at revision, so please note any specific errors here.

Reviewer #2: Yes

6. Review Comments to the Author

Please use the space provided to explain your answers to the questions above. You may also include additional comments for the author, including concerns about dual publication, research ethics, or publication ethics. (Please upload your review as an attachment if it exceeds 20,000 characters)

Reviewer #2: I am happy with the revision and only two minors should be revised.

*We thank the Reviewer for her/his approval*

Please improve the quality of Fig 3.

*We thank the Reviewer for her/his comment. We have improved the quality of Fig. 3 by uploading the file to the Preflight Analysis and Conversion Engine (PACE) digital diagnostic tool (https://pacev2.apexcovantage.com/). PACE helps ensure that figures meet PLOS requirements. It is possible to download the corrected figure by following the link in the pdf sent for approval.*

Please explain how the reactor was kept aseptic.

*Thank you for this suggestion. We report the complete sterilizing techniques protocol:*

***Cell Culture & IVTECH Bioreactor***

*The staff who worked on this project always followed all the good standard procedures:*

- *wash hands thoroughly before and after working with cell cultures;*
- *wear a lab coat, gloves and safety glasses to protect themselves from any hazardous materials and to prevent contamination of the cell cultures from microbes present on skin and clothes.*

***Work area***

- *Static and dynamic cell cultures (IVTECH Bioreactor) were carried out in a biosafety cabinet, under a laminar flow, located in an ad hoc cell culture room.*
- *The inside of the biosafety cabinet was wiped with disinfectant (benzalkonium chloride solution) followed by 70% ethanol before and after use. Further, UV light was turned on to sterilize the biosafety cabinet when unused.*
- *Areas of work only contained the items required for the current procedures.*

***Handling & sterilizing techniques***

- *70% ethanol was sprayed on gloved hands before starting the work in the biosafety cabinet.*
- *All the equipment and reagents (cell culture media or other reagents) utilized into the biosafety cabinet were sprayed beforehand with 70% ethanol.*
- *Disposable plastic pipettes were used to manipulate solutions, Pasteur glass pipettes were autoclaved as well as each single component of the bioreactor (connector, holder, upper and lower cylinders, top and bottom cylinders, thin glasses reservoirs). Specifically, each component was wrapped in aluminum foil and, after autoclaving, all the packages were opened into the hood and the bioreactor circuit was assembled in the hood itself under laminar flow.*
- *The sterilization of the polyester membranes was achieved by keeping them in ethanol 70% for two hours followed by UV light exposure for 15 min.*
